# Expression of p-AKT characterizes adenoid cystic carcinomas of head and neck with a higher risk for tumor relapses

**DOI:** 10.1186/1746-1596-4-18

**Published:** 2009-06-19

**Authors:** Hans-Ullrich Völker, Matthias Scheich, Annette Berndt, Imme Haubitz, Alexandra Metzger, Hans-Konrad Müller-Hermelink, Ulrike Kämmerer, Melanie Schmidt

**Affiliations:** 1 Institute for Pathology, University Würzburg, Würzburg, Germany; 2 Dept. of Otorhinolaryngology, University Würzburg, Würzburg, Germany; 3 Dept. of Gynaecology, University Würzburg, Würzburg, Germany

## Abstract

**Background:**

Adenoid cystic carcinomas are rare tumors with an indolent clinical course, but frequent local relapses. The identification of tumors with a higher relapse risk seems to be interesting. Hence we investigated parameters of glucose metabolism, which were found associated with poor prognosis in other malignancies.

**Methods:**

Specimen of 29 patients were investigated immunohistochemically with antibodies against p-AKT, TKTL-1 (transketolase-like 1), M2PK (M2 pyruvate kinase), and GLUT-1. Proliferation was investigated by staining with Ki67. The tumors were located at the major or minor salivary glands. Only the typical cribriform subtype was investigated. The initial tumor stage was pT1 or pT2.

**Results:**

Expression of p-AKT was significantly (P = 0.036) associated with a higher relapse risk in multivariate analysis. Low expression of M2PK was non-significantly (P = 0.065) predictive for a higher risk. TKTL-1 and GLUT-1 were expressed in the majority of cases, albeit not associated with relapse risk.

**Conclusion:**

Adenoid cystic carcinomas positive for p-AKT show a higher relapse risk. However, other parameters of glucose metabolism investigated here or proliferation (Ki67) were not predictive in this entity. Our findings demonstrate a possible background for therapeutic approaches targeting the inhibition of PI3K/AKT pathway.

## Background

Malignant tumors of salivary glands represent a small subset of all malignancies. Adenoid cystic carcinomas (ACC) are one of the most common types at this anatomic site. They account for 1% of all malignant head and neck tumors, 10% of all salivary gland tumors, and 22% of malignant salivary gland tumors [1,2]. The majority occures at the minor salivary glands (60%) [3]. From the major salivary glands, the parotid is most often involved. Locations like the nasal cavity and the paranasal sinus are also possible. Typically, ACC show an indolent clinical course with a considerable risk for local relapse (60% of patients, mostly <2 years after primary) and late distant metastases (lung or bone), the latter sometimes several years after first diagnosis with fatal outcome. Standard treatment for ACC is surgery, followed by post-operative radiotherapy [4]. Since tumors show a characteristic perineural spread, the surgical key problem is the complete resection. Therefore, the extend of tumor infiltration is sometimes not apparent, and local relapses may originate from microscopic residual disease.

The therapeutic options have not been improved significantly in recent years despite different studies which investigated the influence of chemotherapy or molecular approaches [3,5,6]. However, there are individual differences concerning the outcome. To estimate prognosis, tumor stage, subtype, and the extend of perineural spread were found to be reliable.

In order to identify additional predictive parameters by immunohistochemical investigation, we performed the following study. The intention herein was to clarify the predictive potential of the expression of several markers which are assumed to play an important role in the glucose metabolism, especially phosphorylated AKT (p-AKT), transketolase-like-1 (TKTL-1), M2 pyruvate kinase (M2PK), and glucose transporter (GLUT-1). Their expression was found associated with a poor prognosis in different malignancies [7-15]. To our knowledge, these parameters were not investigated in ACC up to now. The idea for this study is based on the theory of Otto Heinrich Warburg (1883–1970) from 1924, which implies the increase of glucose utilization in all malignant tumors [16].

## Methods

Paraffin embedded tumor specimens of 29 patients (16 female, 13 male) with ACC were included in this study. The locations of primaries are indicated in table 1. In cases with relapses, primaries and their relapses were examined. The time of follow up observation was at least five years. The initial therapy was surgical with consecutive local radiation in all cases. Tumors investigated in this study had a initial tumor stage of pT1 or pT2, N0, M0.

**Table 1:** Site of primary tumors.

**Site**	**n = 29**	**%**
Glandula parotidea	4	13.8

Glandula submandibularis	5	17.2

Glandula sublingualis	1	3.5

Minor salivary glands	10	34.5

Nasal cavity and paranasal sinus	9	31.0

Three histological subtypes of ACC exist (cribriform, solid, tubular). Due to the known differences in prognosis [2], only cases with the typical cribrifom pattern of growth and typical immunophenotyp (Vimentin +, CD117 +) were included. Diagnoses were validated by two experienced pathologists.

### Immunohistochemical staining

Immunohistochemical staining with commercially available antibodies were done in standard technique following manufacturer's protocols. Positive controls were used in accordance with manufacturer's recommendations or own established/published experiences. Antibodies including provider, dilution and positive controls are listed in table 2. All sections including positive controls were stained simultaneously for the appropriate antibody.

**Table 2:** Used antibodies, source and dilution.

**Antibody**	**Clone/species**	**Source**	**Dilution**	**Positive control**
p-AKT	ab28821 polyclonal rabbit	Abcam, Cambridge, UK	1:100	glioblastoma multiforme

TKTL-1	JFC12T10 monoclonal mouse	Linaris, Wertheim, Germany	1:200	colorectal adenocarcinoma

M2PK	DF4	ScheBo Biotech, Gießen, Germany	1:250	colorectal adenocarcinoma

GLUT-1	polyclonal rabbit	DAKO, Hamburg, Germany	1:100	epidermis

Ki67	MIB-1	DAKO, Hamburg, Germany	1:200	tonsil

Slides with tumor sections with a maximum thickness of 2–5 μm were deparaffinized with xylene. Rinsing the slides in decreasing concentrations of ethanol was followed by antigen unmasking in 10 mM sodium citrate buffer (pH = 6.0) in a microwave oven at 600 W for 5 minutes. After rinsing in distilled H_2_O, endogenous peroxidase was inhibited by incubation for 10 minutes in 3% H_2_O_2 _in methanol. Slides were then washed with PBS and incubated with 1% goat serum in PBS for 15 minutes. Subsequently, slides were incubated with the respective primary antibodies diluted in antibody diluent (DAKO, Hamburg, Germany).

After 45–60 minutes of incubation at room temperature, slides were washed in PBS, incubated with an appropriate biotinylated secondary antibody (DAKO), washed and incubated with streptavidin-peroxidase (DAKO). Staining was visualized by adding 3.3'-diaminobenzidine (DAB; DAKO) with subsequent counterstaining using haematoxylin. Sections were dehydrated in graded ethanol and embedded in Vitro Clud (Langenbrinck, Germany).

### Statistical analysis and clinical correlation

**Figure 1:**
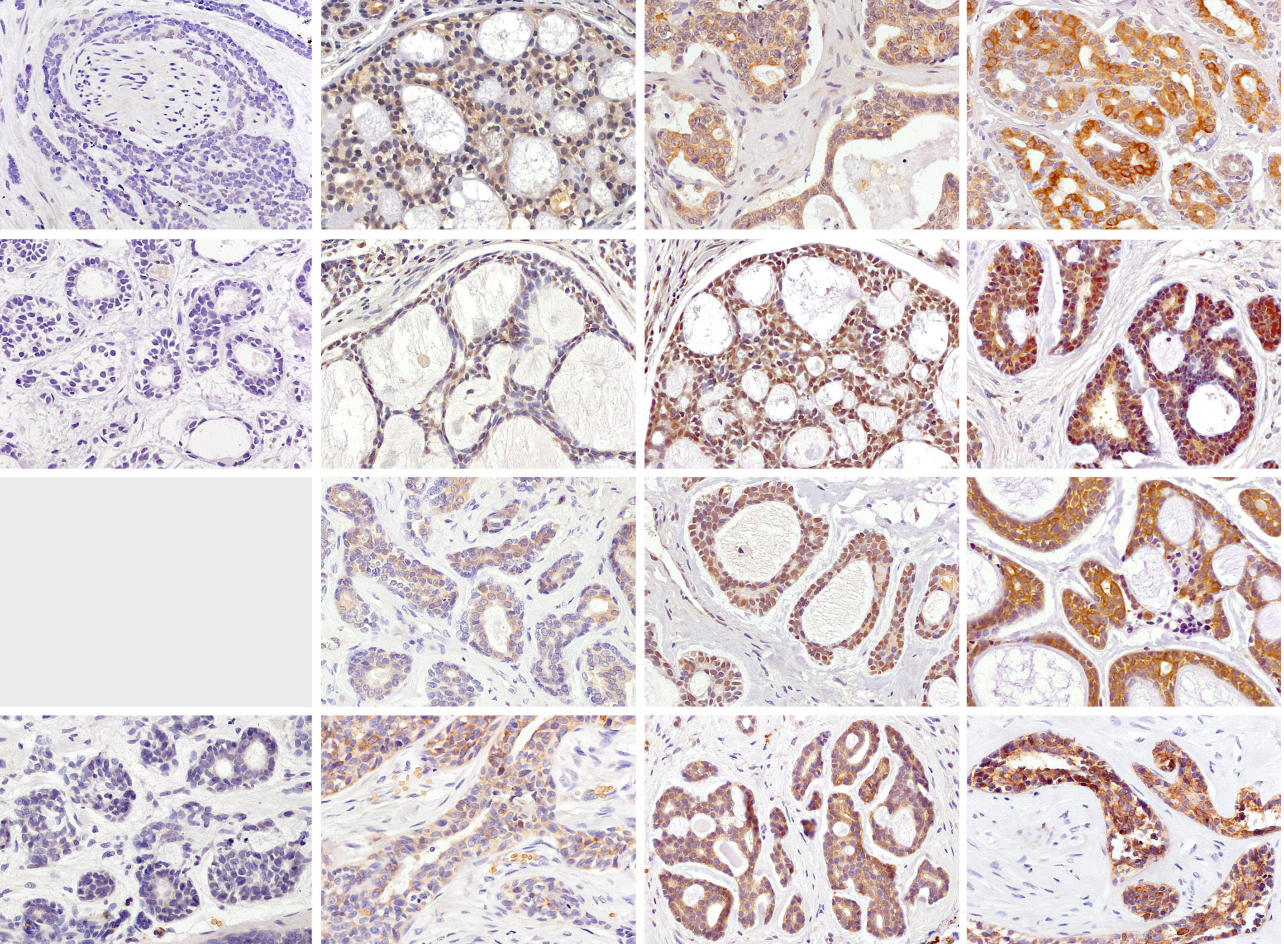
**Examples for immunohistochemical staining results: row 1: p-AKT, row 2: TKTL-1, row 3: M2PK, row 4: GLUT-1; column 1: negative (no negative case was found when M2PK was stained), column 2: weak, column 3: moderate, column 4: strong**.

Staining intensities were scored independently by two observers with experiences in immunohistochemical investigations. Differences were discussed (no differences between negative or positive, but some between low and moderate staining intensity). Scoring grades were 0 (negative), 1 (low), 2 (moderate) and 3 (high staining intensity). Examples are shown in Figure 1.

Statistical tests were done using the SPSS software package (version 14.0.1, Chicago, IL). Charcteristics are presented at means with their SD values for normally distributed variables. The significance level was set at α = 5% for all comparisons. Significances were assessed with log-rank statistics, Kendall rank correlation, U-Test by Mann and Whitney, or chi-square test. The cox proportional hazards model was used for the multivariate analysis.

### Ethics

The ethic approval for this study was obtained from Ethic Committee of the Medical Faculty of University Wuerzburg. The study was performed in accordance to the Helsinki declaration.

## Results

**Figure 2:**
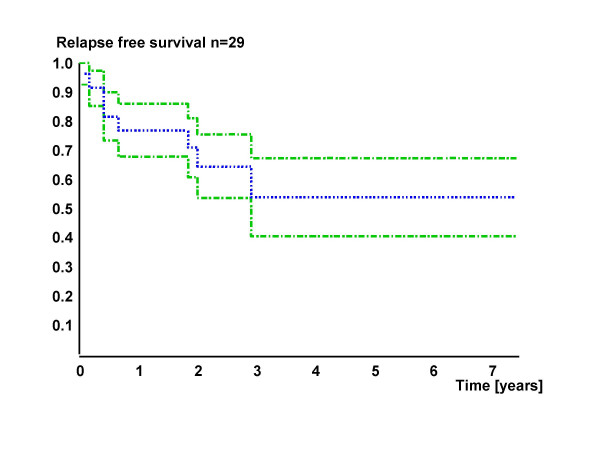
**Relapsing course (overall) with 95% confidence interval**.

Patient's median age was 58 years (±15, 32–86 years). 16/29 (55.2%) of patients were free of relapses after initial surgery. The remaining patients developed relapses within 2–94 months after initial surgery (median 35 months). Figure 2 shows the overall relapsing course. If relapsing, 6 patients had more than one relapse (2–4). The site of primary and risk for relapse did not significantly correlate in this cohort (P = 0.1). However, 70% (7/10) of tumors located in the major salivary glands were disease-free after initial therapy, whereas the majority of relapses arose from tumors of other sites (76.9%). The tumor stage did not influence the risk for relapse in this study.

Comparing the staining results of primaries and relapses of each single patient, no significant differences were found (P > 0.8, U-Test by Mann and Whitney). Therefore, in the following the overall staining results are reported.

**Figure 3:**
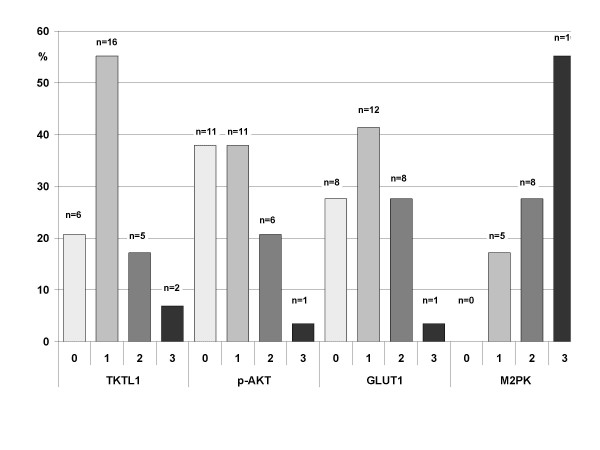
**Results of immunohistochemical staining results in 29 adenoid cystic carcinomas**. Staining intensity (Score): 0-negative, 1-low expression, 2-moderate expression, 3-strong expression

The proliferation (Ki67) reached from <10% up to 50% in primaries and relapses. The differences were not significant and did not show a trend to higher proliferation in relapsing cases. Figure 3 shows the results and distribution of immunohistochemical staining. Significant correlations of different staining results are given in table 3. Interestingly, elder patients showed a significant lower expression of M2PK (tau = -0.28, P = 0.031, Kendall rank correlation). For the different sites, tumors of nasal cavity or paranasal sinuses stained weaker for TKTL-1 than others. Staining intensities were distributed equally in all tumors with 80–100% positive tumor cells. A focal staining was not observed.

**Table 3:** Correlation between different staining results (Kendall rank correlation).

**Stainings**		**tau**	**P**
p-Akt	TKTL-1	0.3848	0.0034

p-Akt	M2PK	0.4015	0.0022

TKTL-1	Glut-1	0.2650	0.044

TKTL-1	M2PK	0.5395	<0.00005

The multivariate analysis for the predictive role of different factors regarding the relapse risk is indicated in table 4. Apart from well known parameters (status of complete surgical resection), expression of p-AKT was significantly (P = 0.036) associated with a higher relapse risk. Furthermore, lower expression of M2PK was a non-significant (P = 0.065) predictor for a higher relapse risk.

**Table 4:** Multivariate analysis for relapse risk revealed expression of p-AKT and incomplete resection as prognostic parameters.

	**beta**	**SD (β)**	**HR**	**68%-CI von HR**		**P (Chi square)**
**incomplete resection**	12.830	6.505	max	558.725	max	**0.049**

**TKTL-1**	1.264	0.909	3.538	1.426	8.781	0.16

**p-Akt**	4.858	2.394	128.739	11.754	1410.095	**0.042**

**Glut-1**	1.100	0.880	3.003	1.245	7.244	0.21

**M2PK**	-5.252	2.818	0.005	0.000	0.088	0.062

**incomplete resection**	9.811	5.436	18238.626	79.491	max	0.071

**TKTL-1**	1.255	0.865	3.506	1.476	8.328	0.15

**p-Akt**	4.188	2.162	65.911	7.588	572.488	0.053

**M2PK**	-4.400	2.508	0.012	0.001	0.151	0.079

**incomplete resection**	5.962	2.814	388.324	23.289	6474.919	**0.034**

**p-Akt**	2.662	1.167	14.331	4.461	46.032	**0.023**

**M2PK**	-2.092	1.132	0.123	0.040	0.383	0.065

**incomplete resection**	2.713	1.236	15.080	4.380	51.916	**0.028**

**p-Akt**	1.109	0.529	3.032	1.787	5.143	**0.036**

## Discussion

Since Otto Warburg has published his hypothesis of the importance of glucose metabolism in tumor cells, many studies have been published regarding this issue. The influence of PI3K-AKT pathway, glucose transporter GLUT-1 and M2 pyruvate kinase on glycolysis was demonstrated in several investigations [11,12,15]. In addition, transketolase-like-1 (TKTL-1) was postulated to play also an important role in the glucose metabolism of tumors [17]. To our knowledge, this is the first study which elucidates the role of the markers mentioned above in adenoid cystic carcinomas (ACC) of head and neck.

All markers were expressed in ACC of our cohort. In all tumors, 80–100% of tumor cells were positive stained. Therefore we did not respect the percentage of positive cells in the analysis. However, in other tumors we found a more heterogeneous distribution of staining. The cause could be the more homogeneous tumor differentiaton in cribriforme ACC compared e.g. with squamous cell carcinomas or glioblastomas [7,8].

The expression of p-AKT was found as an independent parameter for higher relapse risk in ACC, apart from well documented predictors like incomplete surgical resection, which we also found correlated significantly with higher potential for relapsing. Activation and phosphorylation of the Akt/protein kinase B (AKT) by phosphatidylinositol 3-kinase (PI3K) often appears in malignant tumors [11,18]. Phosphorylated AKT leads to increasing proliferation, tumor growth and decreasing apopotosis as well as stimulation of aerobic glycolysis in tumor cells. Elstrom et al. suggest that the activation of AKT could be responsible for the metabolic processes during the so-called Warburg effect [8,11,18]. One possible reason for AKT activation in ACC was shown by Hu et al., who found an expression of epiregulin (a member of epidermal growth factor family) in ACC cell lines with a higher metastatic risk [19].

However, the expression of other parameters investigated in this study were not significantly associated with higher relapsing potential, despite the fact that they were found associated with worse clinical outcome in other malignant tumors.

TKTL-1 was postulated to be one of three isoforms of the thiamine-diphosphat dependent enzyme transketolase, which plays an important role in the pentose phosphate pathway (PPP) of anaerobic glycolysis [17]. This oxygen-independent pathway serves as an important source for the generation of NADPH_2 _needed in reductive biosynthesis (for example fatty acid synthesis), but more important is its role in nucleic acid ribose synthesis utilizing glucose carbons. More than 85% of ribose recovered from tumor cells is descended from this pathway. Therefore, transketolase upregulation in tumor progression seems very likely. We could show an expression of TKTL-1 in ACC as well as in some other malignant tumors [7,8,10]. However, the expression was not associated with relapse risk or survival. In other studies, tumors with an aggressive clinical course were investigated (e.g. glioblastomas [8]), whereas ACC grows comparatively slow. Only few ACC showd a strong expression of TKTL-1, more often the expression was only weak. With the background of the importance of the PPP in nucleic acid ribose synthesis, our findings fit with the slow growth of ACC. In accordance with the present results, we have not found a correlation between TKTL-1 expression and prognosis in tumors of lower malignancy like astrocytomas <grade IV or granulosa cell tumors of ovary [8,9].

The non-predictive value of M2PK and GLUT-1 can be assumed in the same context. The glycolytic M2 pyruvate kinase isoenzyme (M2PK) plays a key role by channelling glucose carbons either into synthetic processes or towards glycolytic energy production, the former reactions being catalyzed by the dimeric form of M2PK, the latter by its tetrameric form. The dimeric form of M2PK is characteristic for tumor cells [12] resulting in an accumulation of glycolytic metabolites, which are canalized into synthetic processes such as amino acid production or nucleic acid synthesis via the pentosephosphate cycle. Despite non-significant results, similar to other tumors a lower degree of M2PK expression tends to be an unfavorable parameter for prognosis. Interestingly, in our cohort elderly showed a lower expression, but this result was not found associated with another relapse risk in this subgroup. The expression and functionality of glucose transporters (GLUT) in tumors is probably related to a number of factors. In this context, the role of oncogenes and tumor hypoxia has generated a considerable interest [20]. In squamous cell carcinomas and other malignancies, hypoxia induces a higher expression of GLUT [21]. The expression of GLUT-1 was found in several malignant tumors, but not always in correlation with prognosis [9,13-15]. In our study, GLUT-1 was expressed in the majority of ACC, however the expression was mostly weak. Therefore we assume that hypoxia may play an minor role in progression of ACC. Further investigations regarding this point seems necessary (e.g. investigation of hypoxia inducible factor HIF-1α and correlation with GLUT and a prospective study of GLUT function in ACC with 18-FDG-PET).

Due to the missing significance of TKTL-1, M2PK and GLUT-1, an influence of a glycolytic phenotype is not proved as parameter of the tumor progression of ACC. However, further studies with other markers seem necessary. Nevertheless, there was a correlation between expression of p-AKT and TKTL-1 as well as M2PK, so that the connection between these parameters reported in the literature seems once more plausible [8].

## Conclusion

Expression of p-AKT is associated with a higher relapse risk in adenoid cystic carcinomas of salivary glands. In absence of other predictive parameters apart from tumor subtype and status of resection, the immunohistochemical staining could help to assess the individual risk for a more aggressive course of disease. Moreover, our observation is in particular important, hence investigations and clinical trials regarding the therapeutic target of the PI3K-AKT pathway are ongoing [22,23].

## Competing interests

The authors declare that they have no competing interests.

## Authors' contributions

HUV: Main investigations, idea, and discussion; HKMH: Diagnoses; MaS, AB, AM: Clinical data, material, results; UK, MeS: Methodical approaches, theory, discussion; IH: Statistics. All authors read and approved the final manuscript.
